# Get Back in De Ribber

**Published:** 1890-12

**Authors:** 


### GET BACK IN DE RIBBER.

The prevailing high waters and the danger of a sweeping flood recalls to mind a humorous incident of the great flood of 1882, which is good enough to repeat. A certain boat coming up the Mississippi lost her way and bumped up against a frame house. She hadn't more than touched it before an old darkey rammed his head up through a hole in the roof where the chimney once came out and yelled at the captain on the boat: "Whar de hell is you going wid dat boat? Can't you see nuffin? Fust thing yer knows yer gwine to turn dis house ober, spill de old woman an' de chil'en out in de flood an' drown 'em. Wat yer doin' out here in de country wid yer dam boat any how? Go on back yonder froo de co'n field and get back into the ribber where yer b'longs. Ain't got no business sev'n miles out in de country foolin' roun people's houses no how!"

